# LSD2 Is an Epigenetic Player in Multiple Types of Cancer and Beyond

**DOI:** 10.3390/biom14050553

**Published:** 2024-05-03

**Authors:** Hyun-Min Kim, Zifei Liu

**Affiliations:** Division of Natural and Applied Sciences, Duke Kunshan University, Kunshan 215316, China

**Keywords:** LSD2, KDM1B, AOF1, LSD1, KDM1A, histone demethylase, cancer, DNA repair

## Abstract

Histone demethylases, enzymes responsible for removing methyl groups from histone proteins, have emerged as critical players in regulating gene expression and chromatin dynamics, thereby influencing various cellular processes. LSD2 and LSD1 have attracted considerable interest among these demethylases because of their associations with cancer. However, while LSD1 has received significant attention, LSD2 has not been recognized to the same extent. In this study, we conduct a comprehensive comparison between LSD2 and LSD1, with a focus on exploring LSD2’s implications. While both share structural similarities, LSD2 possesses unique features as well. Functionally, LSD2 shows diverse roles, particularly in cancer, with tissue-dependent roles. Additionally, LSD2 extends beyond histone demethylation, impacting DNA methylation, cancer cell reprogramming, E3 ubiquitin ligase activity and DNA damage repair pathways. This study underscores the distinct roles of LSD2, providing insights into their contributions to cancer and other cellular processes.

## 1. Introduction

### A Historical Overview of Research on LSD2

Histone demethylases can be classified into two families based on their enzymatic action: amine oxidase demethylases (LSD1/KDM1A and LSD2/KDM1B) and Jumonji C (JmjC) family [1,2,3,4,5]. The first histone demethylase to be discovered was lysine-specific demethylase 1 (LSD1), which is a homologue of flavin-containing amine oxidases [6,7]. LSD1, also known as KDM1A, and LSD2 are members of the KDM1 family, both playing crucial roles in histone demethylation and epigenetic regulation. While LSD1 and LSD2 share similarities, such as their involvement in histone demethylation, there are distinctive features that set them apart.

LSD2, identified as the LSD1 homolog (also known as KDM1B or AOF1), emerged through domain homology searches of genomic databases, representing the second human histone demethylase to be identified [8]. Despite their shared functions in chromatin modification, LSD1 and LSD2 exhibit unique characteristics owing to differences in their domain structures and regulatory mechanisms.

**Figure 1:**
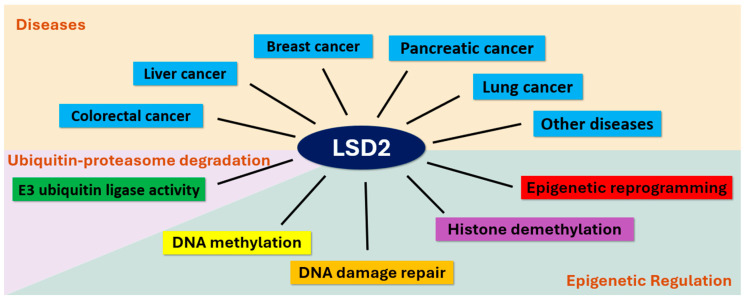
Versatile roles of LSD2 from cancer implications to epigenetic regulation. LSD2 has been implicated in various cancers, either promoting or suppressing human cancers. Additionally, LSD2 is involved in establishing DNA methylation imprints during oogenesis, as well as epigenetic reprogramming during tumorigenesis. Moreover, *C. elegans* LSD2 homolog AMX-1 has been associated with non-transgenerational fertility defects and DNA damage repair. Furthermore, LSD2 is known for its E3 ubiquitin ligase activities, which contribute to the intricate regulation of specific target genes.

Studying LSD2 is important for several reasons. Firstly, it enhances our understanding of the intricate mechanisms governing histone demethylation, contributing to a comprehension of epigenetic regulation. Secondly, as LSD2 is implicated in various human diseases, including cancer, unraveling its complexities may pave the way for targeted therapeutic interventions. Thirdly, the comparison between LSD1 and LSD2 provides a nuanced perspective on the functional diversity within the LSD family. In this paper, we embark on a foundational step in recognizing LSD2’s importance as a key player in cancer and epigenetic regulation, offering potential avenues for therapeutic advancements. We review reports that have investigated cancer tissues and cells, as well as other functions such as DNA demethylation and DNA damage repair (Figure 1).

## 2. Roles and Structure of LSD2 Compared to LSD1

**Figure 2:**
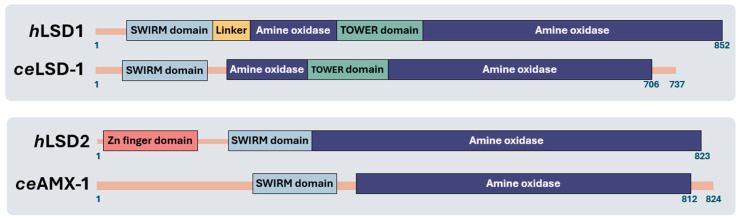
Schematic representations of LSD1 and LSD2. At the top, human and *C. elegans* LSD1 are shown, while at the bottom, the LSD2 homologs are displayed. Most domains are well conserved across the species, except for the zinc finger domain, which is missing in the *C. elegans* LSD2 homolog suggesting a conserved function across species [12,15].

**Figure 3:**
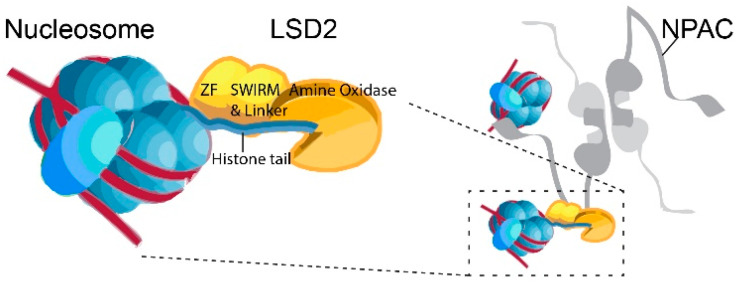
Schematic binding of LSD2 with nucleosome and NPAC. Left: the catalytic cavity in the amine oxidase (AO) domain serves as the first substrate-binding site, binding to the N-terminus of histone H3K4 for demethylation. The linker region forms the second binding site away from the catalytic cavity. This additional interaction is crucial for histone H3 recognition and essential for the demethylation activity of LSD2. Right: NPAC tetramer binds to LSD2-Nucleosome complex. Not all nucleosomes are shown for simplicity purposes. The image was based on the previous reports [11,16].

LSD1 and LSD2 score an overall 33% identity in the SWIRM domain, the FAD coenzyme-binding motif and the C-terminal amine oxidase domains (Figure 2 [6,9]). LSD1 and LSD2 have a conserved SWIRM (Swi3p, Rsc8p, and Moira) domain and an amine oxidase domain (AOD) responsible for their histone demethylase activities [10,11]. However, LSD2 lacks the tower domain, which is instrumental to the LSD1-CoREST association [9,11,12] but possesses a unique C_4_H_2_C_2_-type zinc finger (ZF) and a CW-type zinc finger (ZF-CW) [11,12,13]. The ZF domain in LSD2 is essential for its enzymatic activity, interacting with the SWIRM domain to modulate LSD2 function [13,14]. NPAC/GLYR1, a putative H3K36me3 reader, stabilizes the interaction between LSD2 and H3 peptide, providing an additional layer of regulation to LSD2’s demethylase activity (Figure 3) [11].

The distinct structural organization of LSD2 is reflected in its diverse functional roles. Using ChIP-Chip analysis Fang et al. found that LSD2 predominantly associates with the coding regions of actively transcribed genes, but not the promoters [12]. Furthermore, LSD2 forms active complexes with euchromatic histone methyltransferases G9a and NSD3 as well as cellular factors involved in transcription elongation, proposing that LSD2 and G9a provide additional layers of control to maintain the repressive chromatin structure during elongating chromatin. This control is suggested to be crucial for faithful transcription, however this idea remains to be investigated.

In contrast, LSD1, enriched at promoter regions, contributes to the conversion of mono- or dimethylated histone H3 (H3K4me1/me2) into unmodified H3, influencing various biological processes such as development and tumorigenesis [10]. This divergence in domain architecture and functional roles underlines the specialized contributions of LSD1 and LSD2 to the intricate landscape of histone modifications and epigenetic regulation in cellular processes and disease pathways (Figure 1).

### LSD2’s Enzymatic Activity

LSD2, recognized as a member of the histone lysine demethylase family, actively participates in the dynamic regulation of histone methylation, specifically targeting histone H3K4me1/me2 substrates. The catalytic mechanism of LSD2 involves the flavin adenine dinucleotide (FAD)-dependent amine oxidase domain (AOD) shared with LSD1, as elucidated by Shi et al. in their groundbreaking work in 2004 [6]. This domain facilitates the conversion of mono- or dimethylated histone H3 (H3K4me1/me2) into unmodified H3, thereby influencing chromatin structure and gene expression.

The catalytic AOD serves as the initial binding site for recognizing the N-terminal H3K4me2. Additionally, LSD2 possesses a second binding site located in the linker region, a feature not apparent in LSD1 (Figure 3). The second binding site facilitates the substrate interaction and is essential for demethylation activity of LSD2 [11].

The histone demethylase activity of LSD2 differs from LSD1, relying on a specific linker peptide from the protein NPAC (also known as GLYR1 or NP60, Figure 3) [16]. NPAC serves as a transcriptional co-activator commonly associated with the H3K36me3 epigenetic marks found within gene bodies. While LSD1-CoREST establishes a nucleosome docking platform at silenced gene promoters, LSD2-NPAC forms a complex characterized by flexible linkers, aiding RNA polymerase advancement on actively transcribed genes. The LSD2-NPAC complex plays a crucial role in nucleosome demethylation [17], influencing RNA polymerase progression; its depletion results in downregulated gene transcription. A short sequence (residues 214–225) of NPAC acts as the binding site for LSD2, facilitating the capture and processing of the H3 tail within the nucleosome context. This, in turn, promotes the removal of the H3K4me1/2 marks.

In the examination of the fission yeast *Schizosaccharomyces pombe*, both LSD1 and LSD2 have been implicated in roles dependent on catalytic activity as well as independent of it [18]. Null mutations in Lsd1 and Lsd2 result in either severe growth defects or inviability, while catalytic inactivation causes minimal defects. This suggests that Lsd1 and Lsd2 have essential functions beyond their known demethylase activity. Further exploration has uncovered that an N-terminal peptide is vital for the nuclear localization of Lsd2 and for viability. Additionally, both Lsd1 and Lsd2 participate in the maintenance and establishment of heterochromatin, collaborating with Class II and Sirtuin family HDACs in the constitutive silencing of heterochromatin.

## 3. LSD2 in Human Cancers

LSD2 has been linked to numerous important biological processes including transcription regulation, chromatin remodeling, genomic imprinting, heterochromatin silencing, growth factor signaling and somatic cell reprogramming [12,19,20,21,22]. Also, growing evidence implicates LSD2 in various human diseases, with a significant focus on its role in cancer. Elevated levels of LSD2 have been consistently observed in several cancer types, such as lung cancer, breast cancer, pancreatic cancer, colorectal, and liver cancer.

### 3.1. Breast Cancer Cells

While the activities of LSD1 in facilitating breast cancer progression have been well-characterized, the roles of LSD2 in cancer are relatively less understood. Two reports have elucidated how LSD2 promotes breast cancers; thus, the inhibition of LSD2 attenuates breast cancer progression. Katz et al. reported that inhibition of LSD2 expression leads to accumulation of H3K4me1/2 and attenuates colony formation and downregulates global DNA methylation in breast cancer cells and are more susceptible to cell death [23]. The study also found that combined inhibition of DNA methyltransferase (DNMT) and LSD2 reactivates expression of abnormally silenced genes with important functions in breast cancer and enhances cellular apoptotic responses, suggesting that combinatorial therapy targeting LSD2 and DNMTs effectively improves the antitumor efficacy of DNMT inhibitors in breast cancer.

Later, the same research group showed that the LSD2 protein level was significantly elevated in malignant breast cell lines compared with normal breast epithelial cell line [24]. Also, overexpression of LSD2 in human breast cancer cells altered expression of epigenetic players including LSD1 and HDAC1/2 and promoted colony formation in soft agar assay.

### 3.2. Pancreatic Cancer

Pancreatic cancer is one of the common malignant tumors in the digestive tract with a high fatality rate and is another example where LSD2 is not well studied compared to LSD1. Wang et al. evaluated the functional role of LSD2 in pancreatic cancer cells [25]. LSD2 was highly expressed in pancreatic cancer tissues. Moreover, elevated expression of LSD2/KDM1B was detected in several pancreatic cancer cell lines (BxPC-3, CFPAC-1, PANC-1 and SW1990) as compared with a normal human pancreatic epithelial cell line (HPDE6-C7). LSD2 knockdown inhibited pancreatic cancer cell proliferation and induced the apoptosis of PANC-1 and SW1990 cells suggesting that LSD2 promotes pancreatic cancers.

LSD2 knockdown promotes the activation of pathways involving p-ERK1/2, p-Smad2, p-p53, cleaved PARP, cleaved caspase-3, cleaved caspase-7, p-eIF2a, and survivin. Simultaneously, suppression of IκBα suggests alterations in various signaling pathways associated with various cellular processes such as growth, differentiation, apoptosis, and stress responses, which are influenced by LSD2 expression.

### 3.3. Colorectal Cancer

Colorectal cancer is one of the most common malignant tumors, ranking fourth and second in morbidity and mortality, respectively, among all tumors [26]. While high LSD1 expression has been found in colorectal cancer tissues and inhibition of LSD1 impairs proliferation of colon cancer cells [27], the role of LSD2 in colorectal cancer biology remains relatively not well examined.

Cai et al. showed that LSD2 is upregulated in colorectal cancer tissues [28]. Furthermore, LSD2 overexpression promoted colorectal cancer cell proliferation and inhibited cell apoptosis, while LSD2 knockdown dramatically inhibited the cell cycle by causing G1/S arrest and repressed colorectal cancer proliferation by regulating the p53-p21-Rb pathway both in vitro and vivo. More importantly, LSD2 binds to promoter of p53 and may transcriptionally repress p53 expression via H3K4me2 demethylation.

This suggests an association between LSD2 and the p53 promoter, contrary to reports suggesting LSD2’s enrichment at gene bodies of actively transcribed genes, unlike LSD1 which is known to be enriched at promoters [12]. Reconciliation of these discrepancies could involve comprehensive genome-wide mapping studies using chromatin immunoprecipitation followed by sequencing (ChIP-seq) coupled with functional assays to elucidate the precise mechanisms underlying LSD2-mediated regulation of gene expression. Additionally, exploring context-specific roles of LSD2 under different cellular conditions or in various disease states may provide further insights into its genomic targeting and functional implications.

### 3.4. Lung Cancer Cells

Yang et al. reported that LSD2 is not only a histone demethylase but also functions as an E3 ubiquitin ligase. LSD2 ubiquitylates O-GlcNAc transferase (OGT), leading to the proteasome-dependent degradation of OGT [29]. Interestingly, this LSD2’s E3 ligase activity, not its demethylase activity, played a crucial role in inhibiting the growth of A549 lung cancer cells. Depletion of LSD2 resulted in enhanced colony formation in non-cancerous 293T cells, while the introduction of LSD2 demonstrated an inhibitory effect on the growth of A549 lung cancer cells, emphasizing its E3 ligase-dependent anti-tumor growth function. This study underscores the multifaceted nature of LSD2 as both histone demethylase as well as E3 ubiquitin ligase activities that contribute to the intricate regulation of specific target genes.

### 3.5. Liver Cancer Cells

In a comprehensive study focusing on liver cancer, HCC (Hepatocellular Carcinoma) cell lines were found to exhibit elevated levels of LSD2. Bayo et al. employed various methodologies to identify potential therapeutic targets associated with epigenetic alterations in these cancer cells [30,31]. Previously, it was reported that patients with high tumor expression levels of EZH-2, EHMT2/G9a, KDM3A, KDM4B, and LSD1/KDM1A had significantly worse prognoses [30]. Bayo et al. revealed that higher levels of BRD9, HAT1A, the KMTs (SUV39H2, SMYD1, NSD2, and KMT5A), and the KDMs (LSD1/KDM1A, LSD2/KDM1B, KDM3A, KDM5C, and RIOX2) were associated with poor prognosis among patients with HCC. The study suggested that mutations and alterations in the expression of epigenetic modifiers are common occurrences in human hepatocellular carcinoma, resulting in an aggressive gene expression program and unfavorable clinical outcomes.

## 4. Epigenetic Reprogramming of Cancer

LSD2 is also implicated in reprogramming cancer cells. During hypoxia in glioma-initiating cells (GICs), miR-215 is induced by the HIF-Drosha interaction [32]. This induction of miR-215 leads to the suppression of KDM1B/LSD2, resulting in the alteration of multiple pathway activities and ultimately promoting the progression of GICs within the hypoxic niche. These findings unveil a direct role of HIF in regulating microRNA biogenesis which eventually regulates the KDM1B/LSD2 expression. The identification of the HIF-miR-215-LSD2/KDM1B axis, crucial for GIC adaptation to hypoxia, along with its significant clinical correlation in glioblastoma multiforme patients, may provide an important basis for developing treatments.

In another study by Musella et al., the role of LSD2/KDM1B in cancer reprogramming is reinforced [33]. During immunogenic chemotherapy, IFNs-I act as hubs of resistance, triggering LSD2/KDM1B to facilitate transcriptional reprogramming of cancer cells towards stemness and immune evasion. Consequently, inhibiting KDM1B prevents the emergence of IFN-I-induced CSCs (Cancer Stem Cells). In breast cancer patients undergoing anthracycline-based chemotherapy, KDM1B is positively associated with CSC signatures. Musella et al. identify an IFN-I → LSD2/KDM1B axis as a potent driver of cancer cell reprogramming, advocating for KDM1B targeting as a promising complement to immunogenic drugs to suppress CSC expansion and enhance the long-term efficacy of therapy.

Also, the study by Hou et al. revealed LSD2’s potential for reprogramming cancer [34]. KDM1B enhances somatic reprogramming by inducing the expression of pluripotent genes and promoting cell proliferation. They found that exogenous expression of KDM1B in human dermal fibroblasts can influence the epigenetic modifications of histones. Overexpression of KDM1B can promote cell proliferation, reprogram metabolism, and inhibit cell apoptosis. Additionally, a series of multipotent factors, including Sox2 and Nanog, and several epigenetic factors that may reduce epigenetic barriers, were upregulated to varying degrees. Therefore, it is suggested that LSD2/KDM1B is an important epigenetic factor associated with pluripotency.

## 5. DNA Methylation

LSD2’s roles are not restrained in histone demethylation. Ciccone et al. underscore the significance of H3K4 demethylation in establishing DNA methylation imprints during oogenesis [19]. They demonstrate LSD2’s heightened activity in oocytes, where genomic imprints originate. The study reveals LSD2’s essential role in removing H3K4 methylation from histone H3 and its necessity in initiating DNA methylation on select genes in oocytes. While disruption of LSD2 had no discernible impact on development or oogenesis, oocytes from LSD2-deficient mice exhibited elevated H3K4 methylation and failed to establish DNA methylation marks on four of seven examined imprinted genes. Consequently, embryos derived from these affected oocytes displayed aberrant gene expression patterns and were unable to survive beyond mid-gestation.

## 6. DNA Damage Repair

While LSD2 has primarily been studied for its epigenetic and oncogenic roles, emerging research in model organisms suggests its involvement in DNA damage repair processes.

Recent studies in *C. elegans* have elucidated the roles of LSD2 homolog AMX-1 and its potential implications in DNA repair mechanisms [4,15]. Lack of AMX-1 expression results in embryonic lethality, decreased brood size, and disrupted organization of premeiotic tip germline nuclei. Loss of AMX-1 function activates CHK-1 kinase downstream of ATR, leading to RAD-51 foci accumulation, increased DNA damage-dependent apoptosis, and reduced sensitivity against ICLs (interstrand crosslinks). LSD2/AMX-1 is crucial for the proper expression of mismatch repair component MutL/MLH-1 and sensitivity against ICLs, indicating its involvement in ICL repair and mismatch repair pathways.

Consistent with differences observed in mammalian LSD1 and LSD2, functional disparities were discovered in their *C. elegans* homologs as well. In contrast to LSD1 homolog SPR-5 mutants, which exhibit progressive fertility defects over generations, LSD2/AMX-1 mutants display immediate fertility issues, including reduced sperm count and low brood size or sterility due to failures in germline development. While p53/CEP-1 function is dispensable for sterility in LSD1/SPR-5 mutants, sterility in LSD2/AMX-1 mutants is mediated by p53/CEP-1 function suggesting that sterility of the two histone demethylases is positioned in different pathways. Furthermore, the upregulation of Piwi expression in LSD2/AMX-1 mutants suggest AMX-1’s involvement in regulating germline development and transposon silencing.

In both human cells and *C. elegans* studies, evidence of both redundant and non-redundant functions of LSD1 and LSD2 has been found. Elevated expression of LSD2 leads to an increase in both mRNA and protein levels of LSD1, suggesting potential redundancy between these two histone demethylases. However, concurrent treatment with LSD1 siRNA in both the control group and LSD2-overexpressing cells yields comparable effects on tumor cell growth mediated by LSD2 [24]. This suggests that LSD1 and LSD2 might serve distinct roles in promoting breast cancer proliferation, rather than redundant functions.

In the *C. elegans* model, both redundant and non-redundant roles of two genes were systematically compared. The redundant function was evidenced by observing the compensation of gene expressions between AMX-1 and SPR-5, as demonstrated by quantitative real-time PCR. Additionally, immunostaining results revealed an increase in AMX-1 expression upon lack of SPR-5 in mitotic embryonic cells and gut cells, where the two histone demethylases overlap. However, the absence of SPR-5 did not affect H3K4me2 levels in the premeiotic tip, while elevated H3K4me2 levels were observed at the premeiotic tip in the *amx-1* mutants, suggesting a non-redundant function for the two genes [15].

## 7. Perspectives

LSD2, implicated in various human cancers, holds promise as a potential anti-cancer target. However, its multifaceted roles pose challenges in therapeutic development (Table 1). Understanding its diverse functions, such as gene desilencing and its role as an E3 ubiquitin ligase, is essential for successful clinical translation. Additionally, its involvement in DNA methylation, DNA damage repair, and transgenerational effects further complicates therapeutic strategies. For instance, LSD2’s controversial roles in different tissues, having roles in both promoting and suppressing tumorigenesis, raise questions about its precise therapeutic targeting and highlight the need for comprehensive understanding in therapeutic development (Table 2).

Below are some considerations for studying LSD2.

Epigenetic Crosstalk and Inheritance: Examining the interplay between LSD2 and other epigenetic modifiers, such as histone methyltransferases and chromatin remodelers, may uncover complex regulatory networks governing chromatin dynamics. Given the various roles of LSD2 as a histone modifier and DNA demethylase implicated in oogenesis and cancer, understanding its interactions with other epigenetic factors is crucial for identifying therapeutic strategies for cancer and other diseases.

It is worth noting that while LSD2’s roles in H3K4 demethylation during oogenesis are significant, its homolog in *C. elegans*, AMX-1, does not exhibit distinct transgenerational effects. Exploring these differences across species could provide insights into LSD2-mediated epigenetic modifications and their transgenerational effects.

Therapeutic Targeting of LSD2: Exploring LSD2 as a potential therapeutic target in various cancers holds promise, as evidenced by reports indicating improved antitumor activity in breast cancer with the combination of LSD2 inhibition and DNMT inhibitors [23]. Investigating small molecule inhibitors or other modalities to modulate LSD2 activity may open new avenues for cancer treatment, especially in cases of LSD2 overexpression. However, structural similarities between LSD1, LSD2, and monoamine oxidases (MAOs) present challenges, as inhibitors targeting MAOs may also affect LSD1 and LSD2 [35,36,37,38]. Therefore, developing LSD1/2-specific inhibitors is essential to avoid off-target effects.

SP-2509, an inhibitor of LSD1, has shown potential for specific targeting. It has been demonstrated that Ewing sarcoma cell lines are highly susceptible to a small molecule LSD1 blockade with SP-2509 [39]. However, SP-2509 does not affect LSD2-targeted genes using Gene Set Enrichment Analysis (GSEA). Currently, there are no small molecule agents that specifically target LSD2. Therefore, future research efforts should focus on developing inhibitors tailored specifically to LSD1/2 to address these challenges and further advance cancer therapy.

LSD2 as a Biomarker for Disease Prognosis: Investigating LSD2 expression levels as a potential biomarker for disease prognosis and treatment response could have significant clinical implications. Correlating LSD2 expression patterns with patient outcomes and therapeutic responses in cancer patients may guide personalized treatment strategies, suggesting its potential as a biomarker. For instance, a study by Huang et al. found that PD-L1 was methylated at Lys 162 by SETD7 and demethylated by LSD2, thereby triggering PD-1/PD-L1 interaction [40]. Since PD-L1 K162 hypomethylation contributes to cancer immune surveillance, these findings suggest the potential of PD-L1 K162 hypomethylation as a predictive biomarker for PD-1/PD-L1 blockade therapy response.

Exploring LSD2 in Other Disease Contexts: While research has primarily focused on LSD2’s role in cancer, exploring its involvement in other diseases or physiological processes may uncover additional therapeutic opportunities. This is particularly true, considering that amine oxidases function in various roles including neurons and immune responses. For example, MAOs are located in the outer membrane of mitochondria in neurons and other cells and catalyze the oxidation of monoamines, such as serotonin, dopamine, and norepinephrine, which are neurotransmitters in the nervous system.

Indeed, a search using DisGeNET, a discovery platform of publicly accessible databases of genes linked to human diseases [41,42], found potential associations of LSD2 with types of human diseases other than cancer (Table 3). While 17 cases are associated with various types of cancers, we also found 5 non-tumorous diseases, including atrial fibrillation, familial atrial fibrillation, persistent atrial fibrillation, paroxysmal atrial fibrillation, and Alzheimer’s disease. This further suggests LSD2’s potential contributions to various diseases; however, additional validation is required.

Given the various roles of LSD2, investigating LSD2 in contexts other than cancer may provide novel insights into its biological functions, enriching our understanding of its role in epigenetic regulation and disease pathogenesis. Investigating LSD2’s temporal, spatial, and tissue-dependent roles, along with its differential activities across developmental stages, can provide insights into its context-specific functions.

## Figures and Tables

**Table 1:** Versatile roles of LSD2 from cancer implications to epigenetic regulation.

Findings	Implication
Implicated in various cancers, with roles in promotion or suppression	Human cancer involvement: lung, breast, pancreatic, liver and colorectal cancer tissues or cell lines [23,24,25,28,29,30]
Involved in establishing DNA methylation imprints during oogenesis	Epigenetic regulation during oogenesis [19]
Implicated in non-transgenerational fertility defects in *C. elegans*	Role in fertility defects in model system [4]
Involved in ICL DNA damage repair in *C. elegans*	Role in DNA damage repair in model system [15]
Involved in E3 ubiquitin ligase activities, regulating specific genes	Role in ubiquitin-mediate proteasomal regulation [29]
Implicated in epigenetic reprogramming of cancers	HIF-miRNA-LSD2, IFN-I-induced CSCs and reprogramming through pluripotent gene expression [32,33,34]

**Table 2:** Experimental findings in breast, pancreatic, colorectal, lung and liver cancer associated with LSD2.

Cancer Type	Experimental Results
Breast cancer	Knockdown of LSD2 inhibits breast cancer cell proliferation, migration, and invasion [24].
Pancreatic Cancer	Knock down of LSD2 correlates with cell proliferation decrease and leads to apoptosis increase [25].
Colorectal Cancer	Overexpression of LSD2 leads cell proliferation and apoptosis decrease [28].
Lung Cancer	LSD2 inhibits lung cancer cell growth by promoting OGT degradation [29].
Liver Cancer	Expression levels of LSD2/KDM1B correlated with clinical prognosis of patients [30].

**Table 3:** Diseases associated with KDM1B/LSD2 identified from DisGeNET database.

No	Disease	Disease Class	Semantic Type
1	Atrial Fibrillation	Pathological conditions, signs and symptoms; cardiovascular diseases	Disease or Syndrome
2	Familial atrial fibrillation	Pathological conditions, signs and symptoms; cardiovascular diseases	Pathologic Function
3	Persistent atrial fibrillation	Pathological conditions, signs and symptoms; cardiovascular diseases	Pathologic Function
4	Paroxysmal atrial fibrillation	Pathological conditions, signs and symptoms; cardiovascular diseases	Disease or Syndrome
5	Alzheimer’s Disease	Nervous system diseases; mental disorders	Disease or Syndrome
6	Breast Carcinoma	Neoplasms; skin and connective tissue diseases	Neoplastic Process
7	Malignant Neoplasms	Neoplasms	Neoplastic Process
8	Primary malignant neoplasm	Neoplasms	Neoplastic Process
9	Neoplasms	Neoplasms	Neoplastic Process
10	Malignant neoplasm of breast	Neoplasms; skin and connective tissue diseases	Neoplastic Process
11	Glioblastoma Multiforme	Neoplasms	Neoplastic Process
12	Mammary Neoplasms	Neoplasms; skin and connective tissue diseases	Neoplastic Process
13	Tumor Cell Invasion	N/A	Neoplastic Process
14	Stomach Carcinoma	Digestive system diseases; neoplasms	Neoplastic Process
15	Glioblastoma	Neoplasms	Neoplastic Process
16	Carcinogenesis	Pathological conditions, signs and symptoms; neoplasms	Neoplastic Process
17	Ewings sarcoma	Neoplasms	Neoplastic Process
18	Conventional Renal Cell Carcinoma	Neoplasms; female urogenital diseases and pregnancy complications; male urogenital diseases	Neoplastic Process
19	Pancreatic carcinoma	Digestive system diseases; neoplasms; endocrine system diseases	Neoplastic Process
20	Renal Cell Carcinoma	Neoplasms; female urogenital diseases and pregnancy complications; male urogenital diseases	Neoplastic Process
21	Malignant neoplasm of stomach	Digestive system diseases; neoplasms	Neoplastic Process
22	Malignant neoplasm of pancreas	Digestive system diseases; neoplasms; endocrine system diseases	Neoplastic Process
